# Metabotropic glutamate receptor 5 may be involved in macrophage plasticity

**DOI:** 10.1186/s40659-017-0110-2

**Published:** 2017-02-14

**Authors:** Lali Shanshiashvili, Elene Tsitsilashvili, Nino Dabrundashvili, Irine Kalandadze, David Mikeladze

**Affiliations:** 1 Ilia State University, Tbilisi, Georgia; 2 I.Beritashvili Center of Experimental Biomedicine, Tbilisi, Georgia; 3 Institute of Medical Biotechnology, Tbilisi State University, Tbilisi, Georgia

**Keywords:** Macrophages, Metabotropic glutamate receptors, Inflammation, HMGB1, IL-10

## Abstract

**Background:**

Macrophages are a functionally heterogeneous cell population and depending on microenvironments they polarize in two main groups: M1 and M2. Glutamic acid and glutamate receptors may participate in the regulation of macrophage plasticity. To investigate the role of glutamatergic systems in macrophages physiology, we performed the transfection of mGluR5 cDNAs into RAW-264.7 cells.

**Results:**

Comparative analysis of modified (RAW-mGluR5 macrophages) and non-modified macrophages (RAW-macrophages) has shown that the RAW-mGluR5 macrophages absorbed more glutamate than control cells and the amount of intracellular glutamate correlated with the expression of excitatory amino acid transporters -2 (EAAT-2). Besides, our results have shown that RAW-mGluR5 macrophages expressed a higher level of peroxisome proliferator-activated receptor γ (PPAR-γ) and secreted more IL-10, high mobility group box 1 proteins (HMGB1) and Galectin-3 than control RAW-macrophages.

**Conclusions:**

We propose that elevation of intracellular glutamate and expression of mGluR5 may initiate the metabolic rearrangement in macrophages that could contribute to the formation of an immunosuppressive phenotype.

## Background

Macrophages play an important role in host defense and maintenance of tissue homeostasis. Macrophages are a functionally heterogeneous cell population and depending on microenvironmental stimuli they polarize in two main groups: classically activated macrophages (or M1), whose activating stimuli are interferon-γ (IFN-γ) and lipopolysaccharide (LPS), and alternatively activated macrophages (or M2), which comprise M2a (after exposure to IL-4 or IL-13) and M2c (after exposure to IL-10 or glucocorticoids) cells [1]. Microenvironmental components in blood plasma, including amino acids, can participate in macrophage polarization [2]. During inflammatory states, immune cells release amino acid glutamate (Glu) that induces chemotaxis and regulates endothelial barrier function [3, 4]. Extracellular Glu accelerated cell migration by activating class I/5 metabotropic glutamate receptors (mGluR1/5), expression of which in the macrophages [5], as well as in the microglia has been reported [6, 7]. In these cells, activation of mGluR5 by the selective agonist significantly reduces nitric oxide synthesis [6] and increases the secretion of anti-inflammatory IL-10 [8], suggesting that Glu and mGluR5 may be involved in the activity of an immunosuppressive type of macrophages. Besides, the high intracellular concentration of glutamate can change the redox status and metabolism of innate cells through glutamate/glutamine interconversion and glutathione (GSH) synthesis. The glutamate/glutamine modules play a crucial role in M2 polarization through regulation of TCA cycle [9]. Similar alterations involve during differentiation of tumor-associated macrophages (TAM) [10].

At least two uptake systems are responsible for transporting glutamate into immune cells: the excitatory amino acid transporters (EAAT) and the cystine/glutamate exchanger (xCT). EAAT-mediated glutamate uptake enables a high glutamate concentration gradient to be maintained through the cell membrane, even if extracellular glutamate concentration rises. This gradient stimulates the xc-system and leads to enhanced cystine uptake and GSH synthesis. Activation of EAAT reduced extracellular glutamate levels that may be significant for the prevention of neurological complications, as well as for cancer progression [11]. Macrophages in physiological conditions do not express any of EAAT subtypes, however, in inflammatory conditions LPS and TNF-a increase EAAT expression [12]. It is interesting to note that in reactive astrocytes, acute up-regulation of glutamate uptake through EAAT is mediated by mGluR5a activation [13].

Activation of mGluRs1/5 stimulates intracellular metabolism and gene expression by signaling through the Ras/ERK and PI3K/mTOR pathways. mTOR pathway controls many metabolic processes in immune cells, including macrophage polarization [14, 15]. Among other regulatory proteins, mTOR promotes the expression and activity of peroxisome proliferator-activated receptor γ (PPAR-γ), a master regulator of lipid metabolism [16]. PPAR-y transcriptionally regulates macrophage activation and polarization in health and disease [17]. In addition to the genes, participating in anti-inflammation and lipid metabolism, activation of PPAR-y increases glutamate transporters expression [11, 18].

Among other microenvironmental compounds, high mobility group box 1 (HMGB1) could act as a modulator of macrophage homeostasis [19]. HMGB1 is a highly conserved, non-histone chromosomal protein that play various roles in intracellular and extracellular processes. HMGB1 present within the nuclei and is involved in the maintenance of nucleosome structure and regulation of gene transcription [20]. HMGB1 can also be actively secreted into the extracellular medium by a variety of immune and non-immune cells such as macrophages, monocytes, neutrophils, dendritic cells and natural killer cells in response to various stimuli [21]. Extracellular HMGB1 promotes proliferation, inflammation, energy metabolism, angiogenesis and inhibits host anticancer immunity, apparently through activation of interleukin-1/toll-like receptors (IL-1/TLRs) [22]. Recent investigations have shown that HMGB1 enhances immune suppression through the production of IL-10 by myeloid-derived suppressor cells [23].

Another microenvironmental compound that can modulate the activity of macrophages is galectin-3 (Gal-3). Gal-3 is a β-galactoside-binding lectin of 30 kDa that has been implicated in inflammation and fibrosis [24]. Gal-3 is highly expressed and secreted by macrophages, suggesting its significant role in the innate physiology [25, 26]. Gal-3 expression in macrophages is regulated by cytokines and various components of extracellular milieu. There is some evidence that short-term glutamate treatment of microglia induced a marked increase in galectin-3 release [27].

Taking into account that microenvironmental components modulate macrophage polarization, we hypothesized that glutamate and mGluR5 might be the players in macrophage plasticity. To investigate the role of glutamatergic systems in macrophage activity, we performed the transfection of mGluR5 cDNAs into RAW-264.7 cells. Comparative analysis of these cells has shown that overexpression of mGluR5 in macrophages leads to an elevation in the secretion of IL-10, to the increased expression of PPAR-y and the acceleration of HMGB1 and galectin-3 release. These alterations are correlated with the levels of EAAT-2 and glutamate uptake. Our data suggest that glutamatergic systems and mGluR5 activation may participate in macrophage phenotype transition, probably toward immunosuppressive M2.

## Methods

### Cell culture

Mouse RAW 264.7 macrophages were obtained from the American Type Culture Collection (ATCC) and cultured in plastic cell culture flasks (Greiner Bio One), at 37 °C under 5% CO_2_/95% air in Dulbecco’s Modification of Eagle’s Medium (DMEM; ATCC) supplemented with 10% (v/v) heat-inactivated fetal bovine serum (Sigma), 100 U/ml penicillin (Gibco® by Life Technologies) and 100 μg/ml streptomycin (Gibco® by Life Technologies). RAW 264.7 macrophages were used between passage 5 and 15.

### Cell transfection

Cells were passaged the day before electroporation. They were harvested in the exponential growth phase and centrifuged for 5 min at room temperature. After centrifugation cells were counted and resuspended in the appropriate Gene Pulser Electroporation Buffer (Bio-Rad). For electroporation was used mGluR5 plasmid DNA (from Oxford Genetics, UK) 20 μg/ml. As a reporter, Gaussia Luciferase gene was used. The mixture of cells and DNA was transferred to a cold electroporation cuvette; the cuvette was placed in the ShockPod (Bio-Rad) and 100 μl of the cell mix was then subjected to electroporation using a single 20-ms pulse of 1750 V (RAW 264.7 cells). The transfection was performed using Gene Pulser Xcell Electroporation System (Bio-Rad). After transfection cells immediately plated into pre-warmed media with supplements in a 6-well plate. Results were measured 48 h after transfection. For posttransfection analysis, BioLux Gauassia Luciferase Assay kit (New England BioLabs; UK) and Western Blotting were used.

To confirm that observed plasticity resulted from the overexpression of mGluR5 and was not caused by electroporation, parallel controls for electroporation/transfection were used in each experiment): in the cell culture tubes instead, the plasmid-DNA the same volume of electroporation buffer was added. There were no significant differences between the non-transfected and transfected controls (data not shown).

### Western blot analysis

Following the incubation in the presence or absence of different additions (40 μM Glutamate, 100 ng/ml LPS, 20 nM IL-10), cells were removed from the dishes, washed with PBS and homogenized in 50 mM Tris–HCl, pH 7.4, containing protease inhibitors (1 mM PMSF, 5 mg/ml aprotinin, 5 mg/ml pepstatin A, and 5 mg/ml leupeptin). 50 μg of proteins from the homogenate separated by SDS-PAGE on 15% gels. After electrophoresis, the proteins were transferred onto a nitrocellulose membrane (Mini-PROTEAN® Tetra Handcast Systems, BioRad). After blocking with 5% bovine serum albumin and 0.05% Tween 20 in Tris–HCl buffered saline, the membranes were incubated with the corresponding primary antibodies: anti-EAAT-2 (Abcam), anti-HMGB1 (Santa-Cruz Biotechnology), anti-PPAR-γ (Abcam)) and immunoreactivity was detected by enhanced chemiluminescence autoradiography (ECL kit; Santa-Cruz Biotechnology). Protein concentrations were determined using a BCA protein assay kit (Pierce).

### Nitrite assays

The accumulation of nitrite was used as an index of NO production and inflammatory activation in general. Medium samples from cell cultures were incubated with an equal volume of Griess reagent at room temperature for 15 min and assessed for absorbance at 540 nm. Fresh culture medium served as the blank in all experiments.

### Cytokine and glutamate detection

IL-10, HMGB1 protein, and galectin-3 were measured by ELISA according to the manufacturer’s protocol (Abcam). Plates were read at 450 nm using a microplate reader (Elx808, BioTek). For the measurement of the glutamic acid, Glutamate Assay Kit (fluorimetric, Abcam) was used. Assay procedure was performed according to the manufacturer’s instructions. The fluorescence increase was monitored by using a fluorescence plate reader (Twinkle LB970, Berthold technology) at Ex/Em—530–570 nm. Data are the mean ± SD of triplicate wells.

### Statistical analysis

All data are presented as mean ± SEM. Statistical analysis was performed by one-way ANOVA followed by Scheffe’s post hoc comparison test. P < 0.05 was considered statistically significant.

## Results

### Effect of LPS and glutamate on NO and IL-10 secretion

**Fig. 1:**
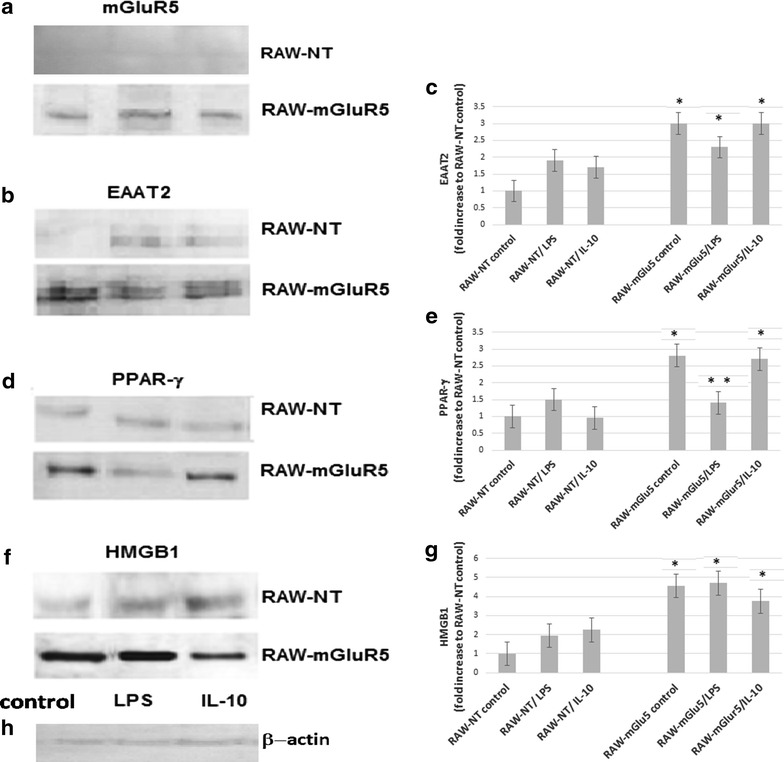
Expression of mGluR5, EAAT-2, PPAR-γ and HMGB1 proteins in control and mGluR5-transfected macrophages. RAW 264.7 cells (RAW-NT) and mGluR5-transfected macrophages (RAW-mGluR5) (5–10^5^ cells per well) were incubated with LPS (100 ng ⁄ ml) or IL-10 (20 nM) for 24 h, followed by the determination of EAAT-2 (**b**), PPAR-γ (**d**) and HMGB1 (**f**) expression by western blot analysis, as described in the “Methods” section. (**h**) β-Actin was also visualized by Western blotting to confirm equal loading of the fractions. Data shown are representative of three independent experiments. Quantification of EAAT2 blots shown in **c**, *P < 0.05, vs corresponding RAW-NT cells. Quantification of PPAR-γ shown in **e**, *P < 0.05, vs corresponding RAW-NT cells, **P < 0.05, vs mGluR5 control. Quantification of HMGB1 blots shown in **g**, *P < 0.05, vs corresponding RAW-NT cells

**Fig. 2:**
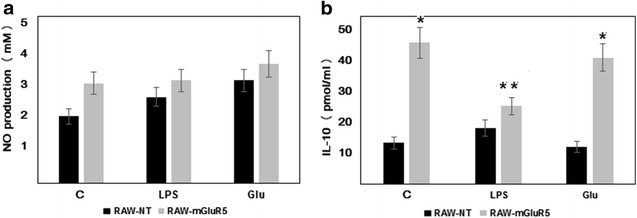
Effect of LPS and glutamate on NO and IL-10 secretion. RAW 264.7 cells (RAW-NT) and mGluR5-transfected macrophages (RAW-mGluR5) (5–10^5^ cells per well) were incubated with LPS (100 ng ⁄ ml) or glutamate (40 μM) for 24 h, followed by determination of NO (**a**) and IL-10 (**b**) secretion, as described in the “Methods” section. Data represented are mean ± SEM of results from four. separate experiments performed in duplicate. *P < 0.05, vs corresponding RAW-NT cells. **P < 0.05, vs RAW-mGluR5 control cells

To clarify the role of mGluR5 in the activity of macrophages, we conducted mGluR5 gene transfection in the murine RAW-264.7 cells. To verify the transfection, western blot of mGluR5 in the RAW 264.7 cells (RAW-mGluR5) were performed. Unlike normal cells, the transfected cells exposed a significant amount of mGluR5 (Fig. 1a) and the level of mGluR5 does not change significantly under the action of the LPS, nor after incubation of cells with IL-10. To characterize these macrophages, at first, we evaluated the ability of cells to release IL-10 and nitric oxide after LPS and glutamate treatments (Fig. 2). We have found that RAW-mGluR5, without LPS stimulation, released more IL-10 (Fig. 2b) than control non-transformed cells, while the amount of secreted nitric oxide did not change significantly (Fig. 2a). 40 μM glutamate that corresponds to a concentration of glutamate in blood plasma has no effect on IL-10 release, secretion of NO or the viability of cells (24 h incubation, data not shown). Therefore, in future experiments, we used the DMEM medium enriched with 40 μM glutamate. The addition of LPS to the non-transfected macrophages did not change the secretion of IL-10, whereas, in RAW-mGluR5, LPS resulted in a decrease in cytokine production. Thus, these data showed that overexpression of mGluR5 in macrophages changes the secretion profile of IL-10.

### Effect of mGluR5 transfection on the expression of EAAT-2 protein

It has been shown that 60–70% of mGluR5 may be localized on intracellular membranes, where they can mediate unique signaling effect [28]. However, the mechanism of the receptor’s activation by intracellular glutamate in macrophages is unknown. It is clear that the activation of this receptor depends on the concentration of intracellular glutamate, which, for its part, is regulated by the glutamate transporters [28]. EAAT2 is the main supplier of glutamate in the macrophages, maintaining the high glutamate gradient across the intra- and extracellular spaces [29]. To assess the role of mGluR5 in the EAAT expression, the level of EAAT2 were determined by western blotting. We have found that transfection of mGluR5 cDNA in macrophages induces the expression of EAAT2 (Fig. 1b, c). However, the significant differences between the effects of IL-10 and LPS are not revealed in the control non-transfected RAW-264.7 cells, because both LPS and IL-10 equally induces the expression of EAAT2.

### Effect of mGluR5 transfection on the glutamate uptake by RAW 264.7 cells

**Fig. 3:**
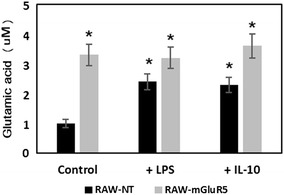
Effect of mGluR5 transfection on the glutamate uptake by RAW 264.7 cells. RAW 264.7 cells (RAW-NT) and mGluR5-transfected macrophages (RAW-mGluR5) (5–10^5^ cells per well) were incubated with LPS (100 ng ⁄ ml) or IL-10 (20 nM) for 24 h, followed by the determination of glutamate in cell lysates, as described in the “Methods” section. Data represented are mean ± SEM of results from four separate experiments performed in duplicate. *P < 0.05, vs RAW-NT control cells

The expression of EAAT-2 could be correlated with the uptake of glutamate by macrophages. Therefore, in the next step, we determined the intracellular concentration of glutamate. Our results have shown that RAW-mGluR5 cells contain more intracellular glutamate than control cells, and the treatment of transformed macrophages with IL-10 or LPS does not change the content of glutamate significantly (Fig. 3). However, both, LPS and IL-10 slightly increase the glutamate uptake in the control non-transfected RAW macrophages. Thus, the amount of absorbed glutamate correlated with the levels of expression of EAAT-2 in macrophages.

### Effect of mGluR5 transfection on the expression of PPAR-γ

Activation of mGluR1/5 stimulates protein synthesis by signaling through the Ras/ERK and PI3K/mTOR pathways. mTOR promotes the expression and activity of various transcription factors, including PPAR-γ, [14] which in turn, can increase functional expression of the EAAT2 [11]. To confirm the hypothesis that in the glutamate-dependent EAAT2 expression, PPAR-γ may be involved, the content of PPAR-γ in the RAW-mGluR5 and RAW-264.7 cells were determined. We have found that normal non-transfected RAW macrophages slightly express the PPAR-γ, whereas the transfection of mGluR5 cDNA in RAW-macrophages greatly increases the expression of PPAR-γ (Fig. 1d, e). It is interesting to note, that in transfected macrophages, LPS reduce the PPAR-γ expression. These data suggest that the expression of EATT could modify the activity of PPAR-γ.

### Effect of mGluR5 transfection on the HMGB1 and Gal-3 secretion

**Fig. 4:**
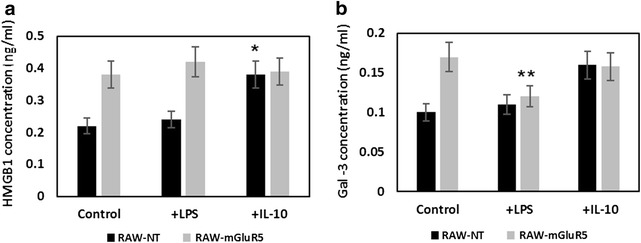
Effect of mGluR5 transfection on the HMGB1 and Gal-3 secretion. RAW 264.7 cells (RAW-NT) and mGluR5-transfected macrophages (RAW-mGluR5) (5–10^5^ cells per well) were incubated with LPS (100 ng⁄ml) or IL-10 (20 nM) for 24 h, followed by the determination of HMGB1 proteins (**a**) and Gal-3 (**b**), as described in the “Methods” section. Data represented are mean ± SEM of results from four separate experiments performed in duplicate. *P < 0.05, vs RAW-NT control cells. **P < 0.05, vs RAW-mGlur5 control cells

PPAR-γ activators exert anti-inflammatory activities in various cell types by interfering with proinflammatory signaling pathways [17]. HMGB1 exhibits a cytokine-like function as a proinflammatory mediator when released from macrophages [21]. HMGB1 stimulated crosstalk between macrophages and myeloid-derived suppressor cells and increased the production of IL-10 [23]. To examine the roles of mGluR5 in the regulation of HMGB1 release, we determined the content of intra- and extracellular HMGB1 in the RAW-mGluR5 and cell culture media after stimulation with LPS and IL-10. We have found that RAW-mGluR5 cells (Fig. 1f, g), as well as culture media of RAW-mGluR5 macrophages, contain a higher level of HMGB1 than control cells (Fig. 4). LPS does not change either intracellular nor extracellular amount of HMGB1 after stimulation of normal cells, whereas IL-10 does not alter the amount of intracellular HMGB1 in both types of cells. IL-10 stimulates the secretion of HMGB1 only in non-transfected cells, whereas in macrophages with overexpressed mGluR5, the sensitivity of cells to IL-10 was slightly dropped in compearison to LPS stimulated RAW-mGlur5 cells (Fig. 4a). Apparently, losing the sensitivity to the IL-10 may be associated with the increased synthesis of PPAR-γ in RAW-mGluR5, what has been described above.

Another cytokine that can be characterized the phenotype of the macrophage is galectin-3. This lectin releases by M2-type macrophages and feedback drives “alternative” macrophage activation [30]. Therefore, modulation of galectin-3 expression during macrophage differentiation may be important in the regulation of macrophage plasticity. To investigate the role of mGluR5 in the galectin-3 expression we treated RAW-mGluR5 and control RAW-macrophages with either LPS or IL-10 and measured the release of galectin-3 in culture media. We found that RAW-mGluR5 released the significantly higher amount of galectin-3 than control RAW-macrophages. In RAW-mGluR5 macrophages the Gal-3 secretion was decreased after treatment of cell with LPS (Fig. 4b). Treatment of macrophages with IL-10 does not change the release of galectine-3 neither in RAW-NT nor in RAW-mGluR5 macrophages.

## Discussion

Macrophages that are present in all tissues are important immune effector cells. Two main phenotypes of macrophages (M1 and M2) induce, suppress, or modulate both innate and adaptive immune responses. These phenotypes play a significant role in the pathophysiology of cancer, autoimmunity and metabolic disorders [31]. M1 and M2 macrophages are determined by the microenvironment and can change in response to new stimuli [32, 33]. Among cytokines and other microenvironmental compounds in the blood plasma, Glu may be one of the modulators of macrophage/microglia plasticity [9, 34, 35]. This amino acid plays a central role in amino acid metabolism, and its concentration in the plasma is regulated between 10 and 50 μM. Elevated plasma Glu has been reported in patients with a various type of pathologies, including malignancy and neurological disorders, whereas low serum Glu was observed in certain autoimmune disorders. It has been proposed that increases in the plasma or serum Glu concentration correlate with an immune deficiency, whereas decreases in plasma Glu concentration display immune hyper-reactivity [34].

The glutamatergic system in macrophages comprises glutamate receptors and glutamate transporters—sodium-dependent EAAT2 and sodium-independent cystine/glutamate antiporter (a system called xc-) [29]. The macrophages, as well as microglia, express mGluR5 [5], which is activated under both normal physiological and pathophysiological conditions [7], however, the role of this system in macrophage/monocyte activation remains to be elicited. It has been reported that selective mGluR5 agonist reduces microglial activation and attenuate the release of pro-inflammatory mediators following stimulation with either LPS or interferon-γ (IFNγ) [6]. Apparently, this effect is due to shifting the balance between M1/M2 microglial activation states towards an M2 phenotype [36]. To clarify the role of the glutamatergic system in macrophages activation, we transfected mGluR5 cDNAs into macrophage-like RAW 264.7 cells. Our results have shown that mGluR5-overexpressed macrophages constitutively release more IL-10 than non-transfected control cells and transfection of mGluR5 cDNA into macrophages does not change the secretion of NO. This data suggests that expression of mGluR5 may contribute, at least in part, to the macrophage polarization towards to the M2 phenotype.

It has been shown that mGluR1/5 can function as an oncogene in certain cell types and that glutamatergic regulation may be significant in tumor progression [37]. The specific targets of mGluR5 in cancer cells, as well as in macrophages/microglia cell lines is not known. Activation of mGluRs1/5 stimulates intracellular metabolism and gene expression by signaling through the Ras/ERK and PI3K/mTOR pathways. Since mTOR pathway controls many metabolic processes in immune cells, including macrophage polarization [14, 15] through the expression and activity of PPAR-γ, we hypothesized that mGluR5 might contribute to the regulation of PPAR-γ expression. Analysis of PPAR-γ in mGluR5-transfected and non-transected macrophages has revealed that overexpression of mGluR5 increases the level of PPAR-γ. These data suggest that mGluR5 may be involved in macrophage polarization through PPAR-dependent transcription systems.

PPAR-γ controls the expression of several genes, including EAAT2 transporter [11]. EAAT2 is the main supplier of glutamate in the macrophages, maintaining the high glutamate gradient across the intra- and extracellular spaces. The Glu transport via the EAATs directly provides intracellular Glu as a precursor for GSH synthesis and assists inward cystine transport via cystine/Glu exchanger (Xc-system) supporting intracellular GSH pool and maintaining intracellular redox balance. In the presence of high extracellular concentrations of glutamate, the cystine/glutamate antiporter functions in reverse, taking up extracellular glutamate and leading to cystine starvation, down-regulation of GSH synthesis and induction of oxidative stress [38]. These changes in redox regulation can alter the polarization and plasticity of macrophages [39]. We have found that transfected RAW-mGluR5 cells express more EAAT2 than control cells and contain a higher amount of intracellular glutamate. These data suggest that mGluR5, through the activation downstream effector systems, may be involved in PPAR-γ-dependent expression of EAAT2, which in turn increases the uptake of glutamate. Elevation of intracellular concentration of glutamate could reverse the cystine/glutamate antiporter functions, leading to cystine starvation, down-regulation of GSH synthesis and induction of oxidative stress that may drive M2 polarization of macrophage [40].

PPAR-y could control macrophage polarization by several pathways. Among other metabolic factors, PPARs induce an NAD+-dependent class III protein deacetylase (SIRT1) gene expression, which increases deacetylation of HMGB1 in the RAW 264.7 macrophages [41]. This modification decreases the LPS-induced release of HMGB1 and changes the inflammatory response of macrophages. On the other hand, HMGB1 increases MDSC (myeloid-derived suppressor cells)—macrophage crosstalk and production of IL-10, thereby skewing macrophages toward a type II tumor—promoting phenotype [23, 42]. Our results have shown that the intracellular level of HMGB1, as well as spontaneous release of HMGB1 from macrophages, increased after transfection of mGluR5. These data suggests that mGluR5-derived HMGB1 may be one of the major players in macrophage-MDSC crosstalk [23].

Growing body of evidence have shown that Gal-3 may be involved in immune tolerance and homeostasis [43]. In alternatively activated immunosuppressive macrophages the level of Gal-3 expression was significantly higher than in classically activated macrophages, suggesting that Gal-3 is a specific and highly upregulated marker of M2-type macrophages. Classical macrophage activation with LPS inhibits galectin-3 expression and release, whereas alternatively macrophage activation by IL-4/IL-13 leads to the accelerated biosynthesis and secretion of galectin-3 [30, 44]. Our data showed that administration of an mGluR5-encoding plasmid into macrophages increased the secretion of Gal-3 that reduces after treatment of cells with LPS. These data once again support the suggestion that overexpression of mGluR5 leads to a transition of the macrophages toward to M2 type.

## Conclusions

In summary, our results suggest that extracellular glutamate and mGluR5 could participate in the plasticity of macrophages by inducing the expression of PPAR-y and EAAT2. The increase in the intracellular concentration of glutamate, for its part, can lead to the rearrangement of oxidative metabolism and accelerate the plasticity of macrophages. Given that mGluR5 transfection induces the release of IL-10, change the secretion of HMGB1-proteins and galectin-3, we propose that glutamatergic regulation of macrophages may participate in M-polarization toward to immunosuppressive phenotype. This type of macrophage plasticity may contribute to the formation of an anti-inflammatory response during tumor development.

## Abbreviations

EAAT-2: excitatory amino acid transporters-2
PPAR–γ: peroxisome proliferator-activated receptor
γ HMGB1: high mobility group box 1 proteins
mGluR1/5: class I/5 metabotropic glutamate receptors
IFN-γ interferon-γ LPS: lipopolysaccharide
TAM: tumor-associated macrophages
xCT: the cystine/glutamate exchanger
TLRs: toll-like receptors
Gal-3: galectin-3
