# Erratum to: Metabotropic glutamate receptor 5 may be involved in macrophage plasticity

**DOI:** 10.1186/s40659-017-0118-7

**Published:** 2017-03-27

**Authors:** Lali Shanshiashvili, Elene Tsitsilashvili, Nino Dabrundashvili, Irine Kalandadze, David Mikeladze

**Affiliations:** 1 Ilia State University, Tbilisi, Georgia; 2 I.Beritashvili Center of Experimental Biomedicine, Tbilisi, Georgia; 3 Institute of Medical Biotechnology, Tbilisi State University, Tbilisi, Georgia

## Erratum to: Biol Res (2017) 50:4 DOI 10.1186/s40659-017-0110-2

In the original version of the article [1], Images 1, 2, 3 and 4 were included as Additional file 1: Figure S1, Additional file 2: Figure S2, Additional file 3: Figure S3 and Additional file 4: Figure S4, respectively. The article has now been updated to include Images 1, 2, 3 and 4 in the main body of the article instead.
